# Synchronous Colonic Adenocarcinoma and Metastatic Lobular Carcinoma in a Colectomy Specimen: A Rare Finding

**DOI:** 10.7759/cureus.3207

**Published:** 2018-08-27

**Authors:** Nektarios Koufopoulos, Christina Goudeli, Eleni Pigadioti, Dimitrios Balalis, Dimitrios K Manatakis, Foteini Antoniadou, Dimitris P Korkolis

**Affiliations:** 1 Department of Pathology, “Saint Savvas” Cancer Hospital, Athens, GRC; 2 Department of Gynecology, “Saint Savvas” Cancer Hospital, Athens, GRC; 3 Department of Surgery, “Saint Savvas” Cancer Hospital, Athens, GRC; 4 Department of Surgical Oncology, “Saint Savvas” Cancer Hospital, Athens, GRC

**Keywords:** lobular carcinoma, metastasis, colon, colorectal carcinoma, gata-3, cdx-2, immunohistochemistry

## Abstract

Invasive lobular carcinoma is the second-most-common subtype of invasive breast carcinoma. Its metastatic pattern is different compared to invasive carcinoma—no special type. It metastasizes more often to the gastrointestinal tract, peritoneum, pleura, and ovaries. The extrahepatic gastrointestinal tract metastases occur mostly in the stomach and small intestine and less often in the colon and rectum.

We present a case description of an 87-year-old woman admitted to our hospital with hematochezia, abdominal discomfort, fatigue, and weight loss. A colonoscopy revealed an exophytic tumor of the sigmoid colon. Metastatic disease was not found in imaging studies. A low anterior resection was performed. The pathologic examination revealed a collision tumor consisting of a poorly differentiated adenocarcinoma of the colon and metastatic lobular carcinoma. The diagnosis was challenging due to the lack of a previous history. Also, the diffuse architectural pattern and signet ring cells found may be in primary signet ring carcinoma of the colon as well as in carcinomas from other anatomical sites. Immunohistochemistry was helpful in making the diagnosis. A review of the literature revealed that this is the fourth case of metastatic breast carcinoma coexisting with colonic adenocarcinoma.

## Introduction

Invasive lobular carcinoma (ILC) is the second-most-common subtype of invasive carcinoma of the breast, accounting for up to 15% of cases. The classical variant displays multifocality, a diffuse architectural, the single-file linear pattern of tumor cells arranged in a concentric or targetoid pattern around normal ducts or structures, and intracellular mucin production either in the form of intracytoplasmic lumina or signet ring cells [1]. Several variants have been reported in the literature besides the classical ILC, including solid, signet ring cell, tubulolobular, alveolar, trabecular, pleomorphic, and a recently described variant with extracellular mucin production [2]. The latter variant shows both intra- and extracellular mucin production, a rare feature among breast carcinomas [3]. The ILC metastatic pattern is different compared to invasive ductal carcinoma, metastasizing more often to the gastrointestinal (GI) tract, peritoneum, pleura, and ovaries [4-5] whereas invasive carcinoma of no special type (NST) metastasizes more often to the lungs, liver, and brain [4]. Extrahepatic GI tract metastasis involves more often the stomach and the small intestine and, very rarely, the colon and rectum [6]. Other rare metastatic sites include the appendix [7], endometrium [8], gallbladder [9], kidney [10], orbit [11], pancreas [12], parotid gland [13], spleen [14], urinary bladder [15], uterine cervix [8], uterus [16], and vulva [17].

We herein describe a case of collision tumor in an 87-year-old woman consisting of a poorly differentiated colonic adenocarcinoma and a metastatic lobular carcinoma as an incidental finding in a colectomy specimen and a review of the literature.

## Case presentation

An 87-year-old patient with no previous history was admitted to our hospital due to hematochezia, abdominal discomfort, fatigue, and weight loss. Colonoscopy showed an exophytic tumor of the sigmoid colon. No evidence of metastatic disease was found on computed tomography (CT) scan or MRI. A low anterior resection was performed.

**Figure 1:**
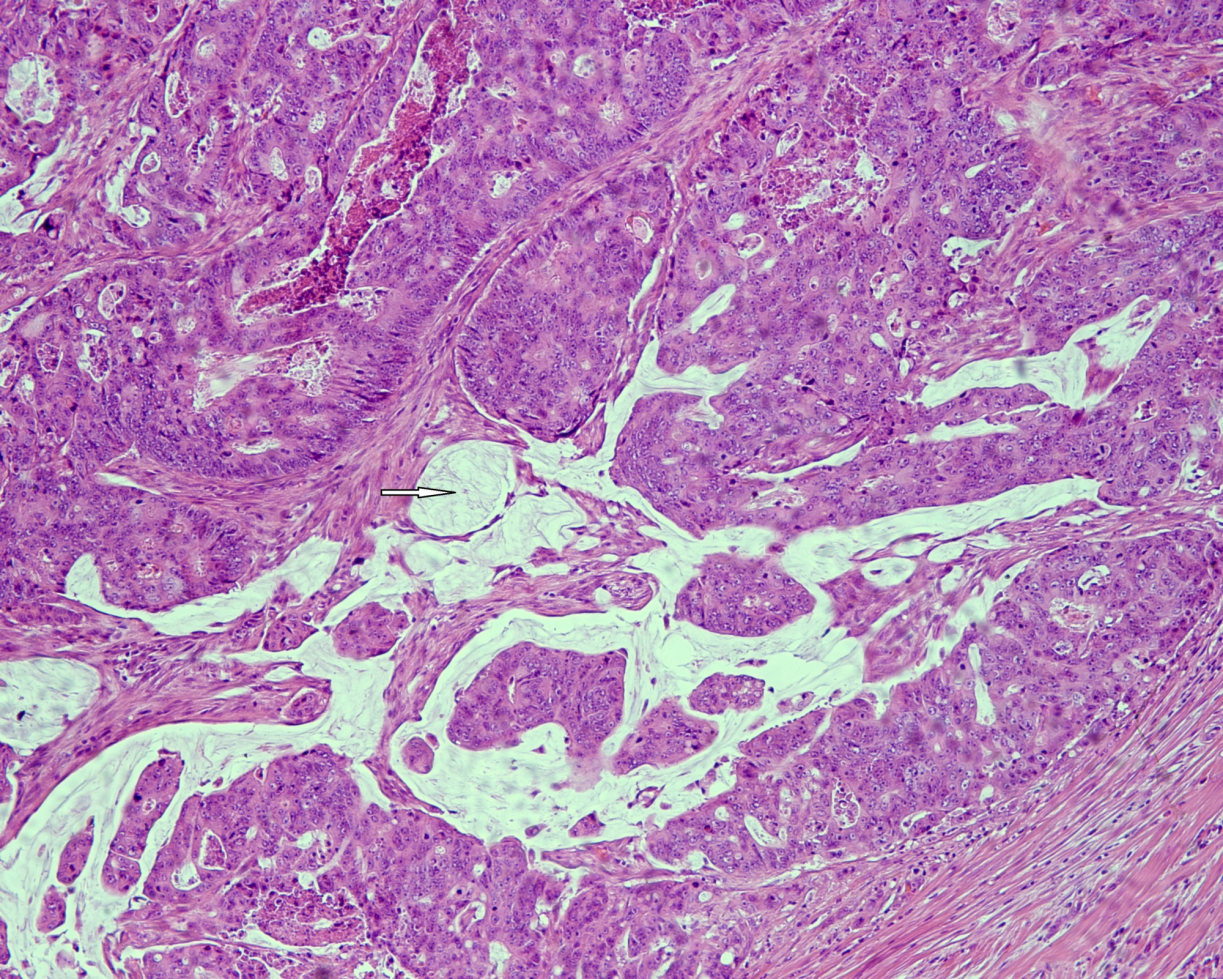
Poorly differentiated colonic adenocarcinoma displaying extracellular mucin production (white arrow) (HE x 100). Abbreviation: HE: hematoxylin and eosin.

**Figure 2:**
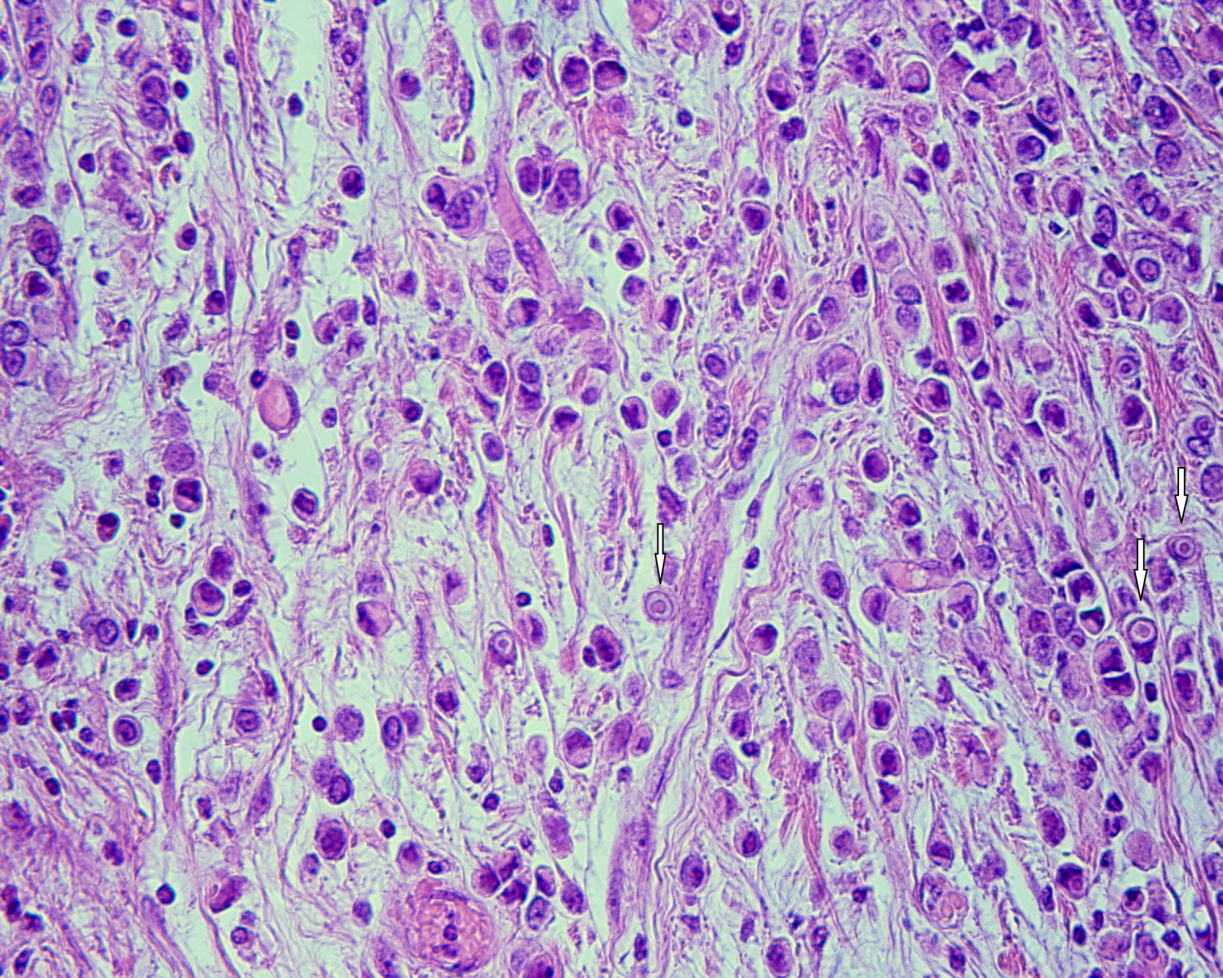
Diffuse type carcinoma cells display eccentric nuclei and intracytoplasmic lumina (white arrows) at high magnification (HE x 400). Abbreviation: HE: hematoxylin and eosin.

**Figure 3:**
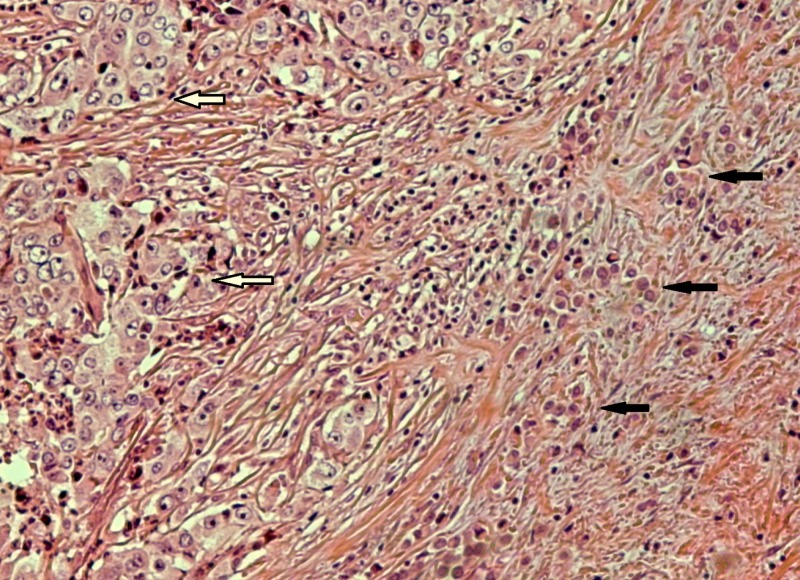
Nests of poorly differentiated colonic carcinoma (white arrows) in close proximity to smaller neoplastic cells (black arrows) with a diffuse architectural pattern (HE x 100). Abbreviation: HE: hematoxylin and eosin.

**Figure 4:**
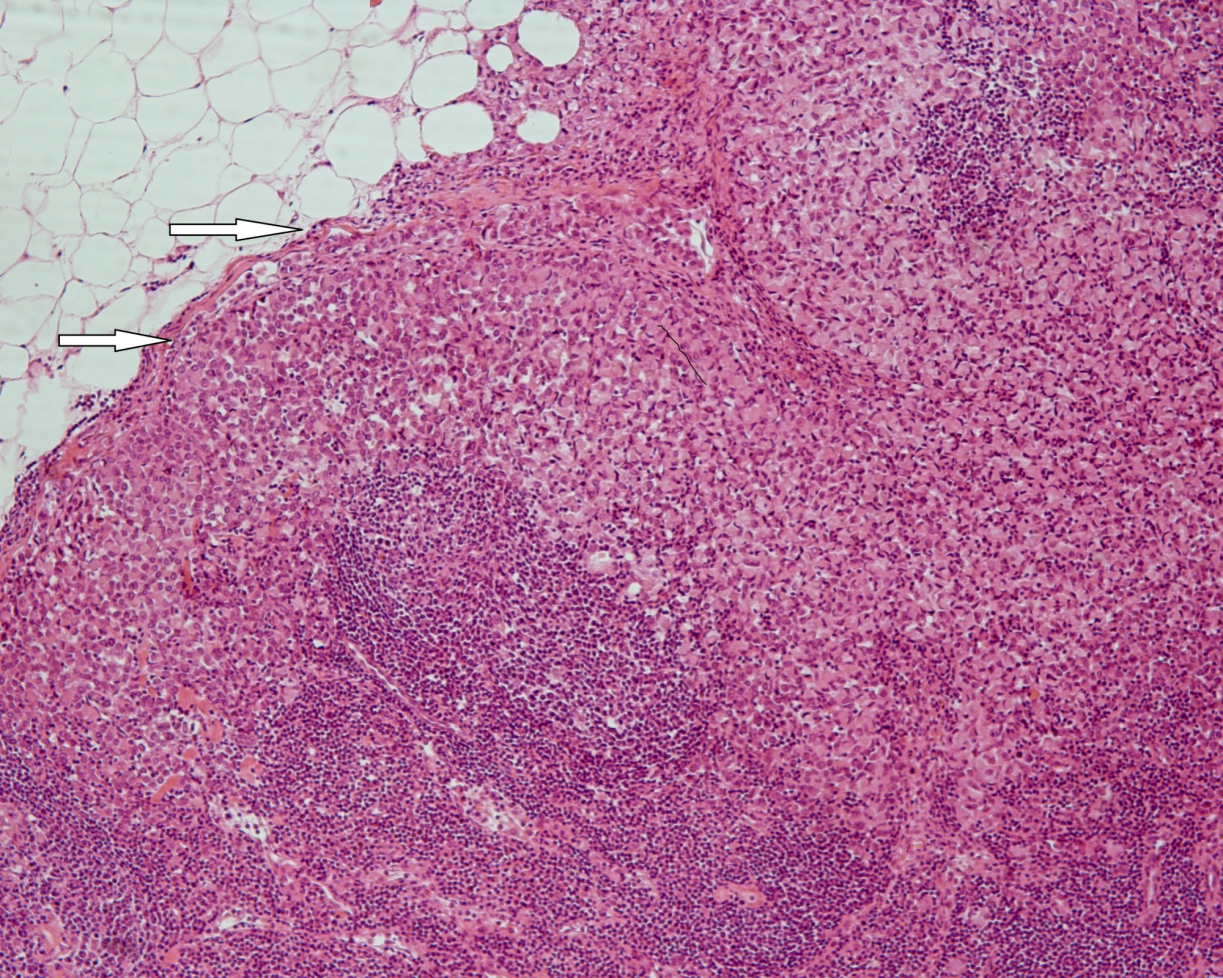
Lymph node infiltration by diffuse type carcinoma cells (white arrows) (HE x 100). Abbreviation: HE: hematoxylin and eosin.

A pathologic evaluation of the resected specimen revealed a poorly differentiated adenocarcinoma of the colon with focal extracellular mucin production (Figure 1) and pericolic fat infiltration. There was a second neoplastic population found mainly near the serosa, consisting of smaller cells with a diffuse architectural pattern, some of them displaying intracytoplasmic lumina (Figure 2). In some areas, both neoplastic populations were found in close proximity (Figure 3). Several signet ring cells were present. Fourteen lymph nodes were infiltrated, five by the colonic carcinoma and nine by the second type of neoplastic cells (Figure 4). Due to the fact that there was no other neoplasm in her history, the second neoplastic population was assumed to be a minor component of primary colonic carcinoma with signet ring morphology. The differential diagnosis also included ILC and other metastatic carcinomas with a diffuse architectural pattern.

**Figure 5:**
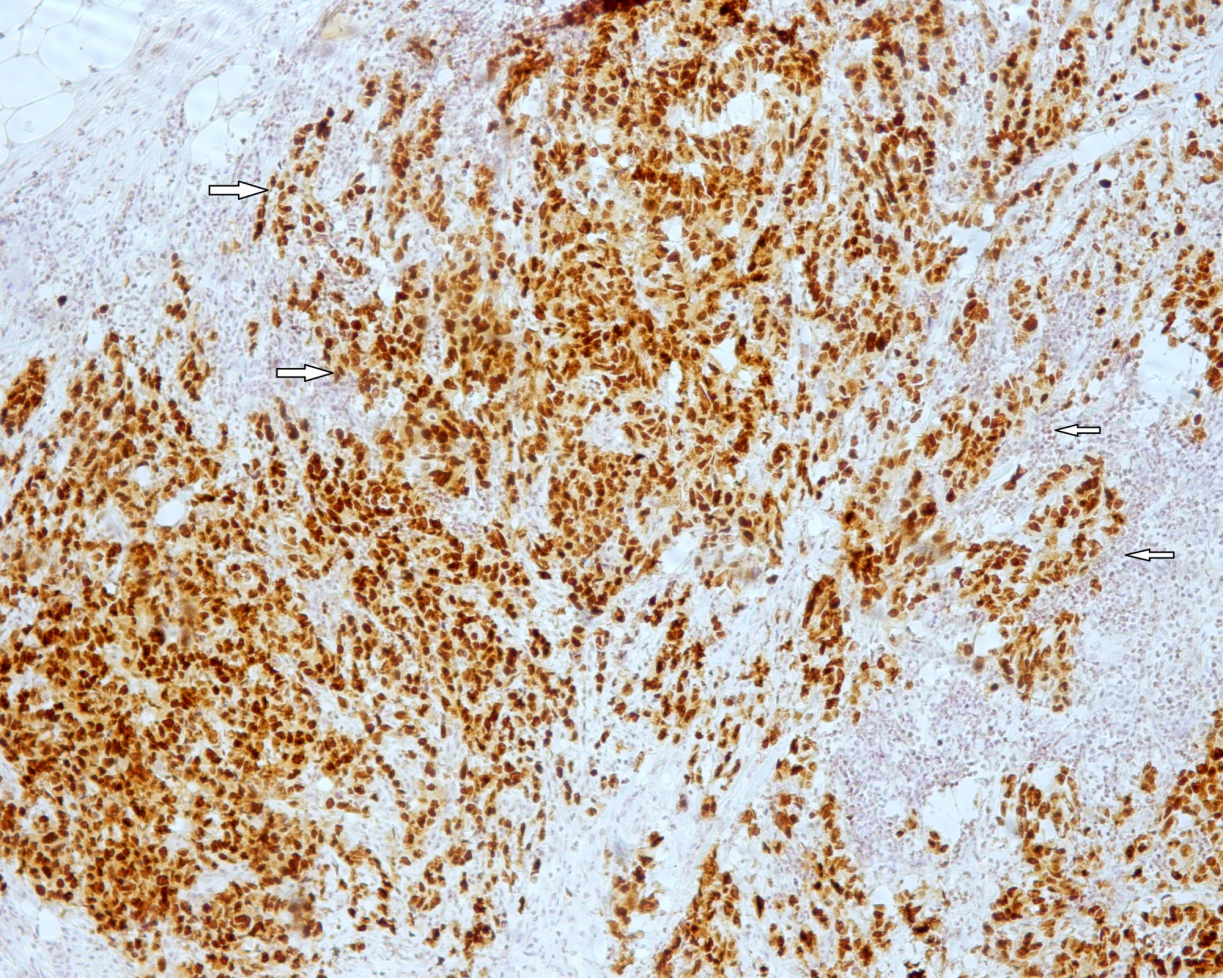
CDX-2 is diffusely positive in colonic carcinoma cells (white arrows) (CDX-2 x 100).

**Figure 6:**
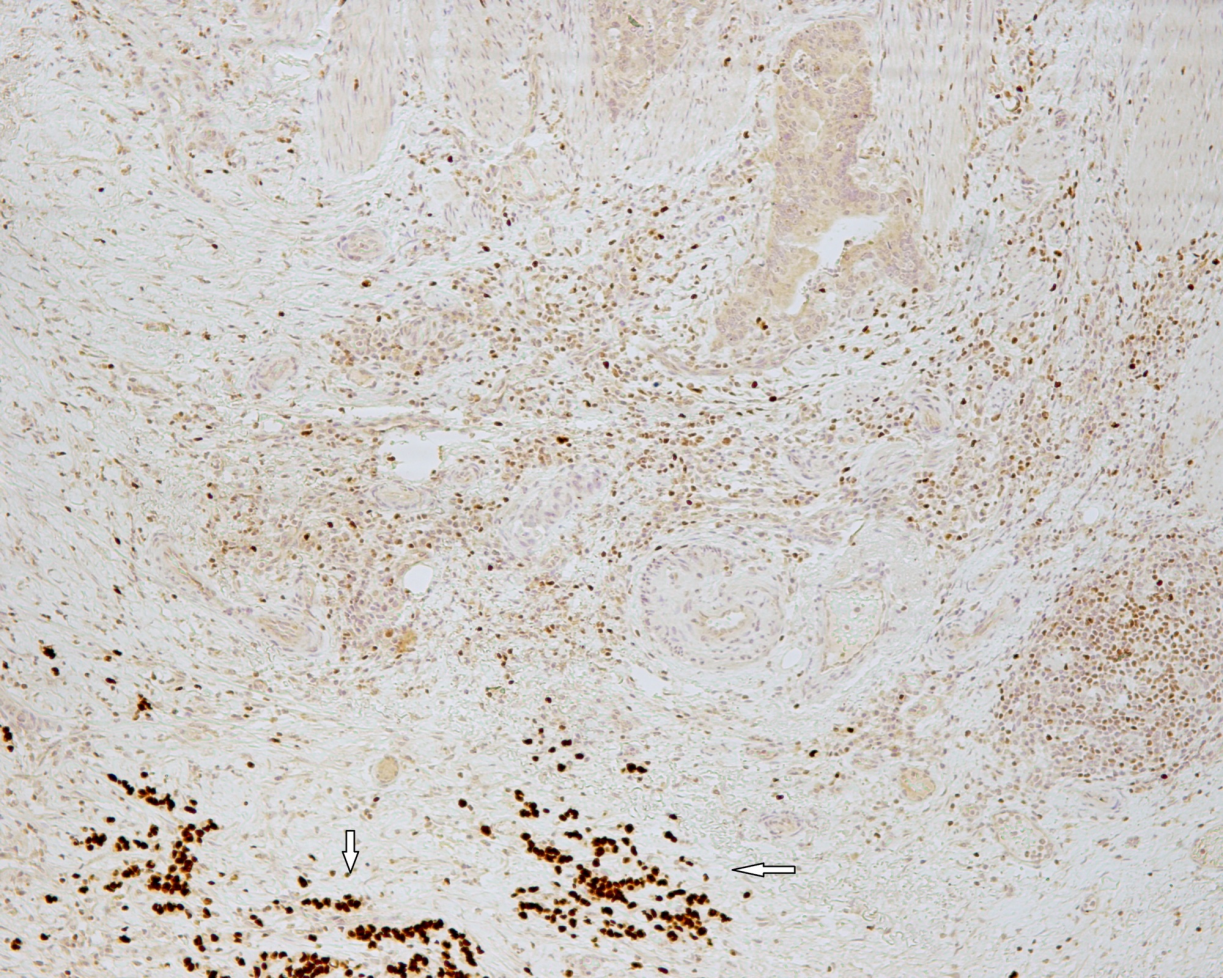
Diffuse type carcinoma cells were positive for GATA-3 (white arrows) while colonic adenocarcinoma cells were negative (GATA-3 x 100).

The immunohistochemical study was negative for CK-20 and CDX-2 in the diffuse type carcinoma cells and positive for them in the colonic adenocarcinoma (Figure 5). CK-7 was negative in both. Additional stainings for GATA-3 (Figure 6), mammaglogin, estrogen receptor (ER)/progesterone receptor (PR) were performed, which were negative in the colonic adenocarcinoma and positive in the diffuse type carcinoma. E-cadherin was negative in the diffuse type carcinoma and positive in the colonic adenocarcinoma. Chromogranin and synaptophysin were negative in both. The diagnosis of a poorly differentiated colonic carcinoma with focal extracellular mucin production co-existing with a carcinoma consistent with metastatic ILC was made.

Imaging studies did not reveal any primary breast tumor. Due to her advanced age, the multidisciplinary tumor board decided to treat her with hormonal therapy. The patient refused further treatment and was lost to follow up.

## Discussion

Breast carcinoma is the most frequent cancer in women, accounting for approximately one-third of cancers, and is a significant cause of morbidity and mortality [18]. GI tract metastases usually occur four to five years after primary breast cancer diagnosis but sometimes 20 or even 30 years later. Occasionally, their presentation is concurrent but, rarely, they may be the first clinical manifestation of breast cancer [4,19]. The rate of GI tract involvement in metastatic breast cancer is around 1% and is found in up to 12% of autopsies [18].

The metastatic pattern of ILC is different from that of an NST carcinoma of the breast. The reason for this different metastatic pattern has been suggested to be the loss of E-cadherin [18]. ILC metastasizes more commonly to the GI tract than NST carcinoma of the breast with an incidence of 4.5% and 0.2%, respectively [18] and is usually associated with disseminated disease [4].

Clinical diagnosis is difficult because colonic metastases may present as a mass mimicking a primary GI tumor, other benign GI tract tumors, or as Crohn’s disease [18,20]. The histological diagnosis may be difficult as well. A previous history of breast malignancy can be very helpful in establishing the correct diagnosis. It has been suggested that for any woman presenting with new GI complaints and a history of breast carcinoma, metastasis must be considered [18]. Attention to histological detail paired with the previous history will usually suffice for diagnosis. In difficult cases, immunohistochemistry will provide the solution as breast carcinoma tumor cells are positive for CK-7, GATA-3, mammaglobin, GCDFP-15, and estrogen and progesterone receptors. In our case, despite the fact that CK-7 was negative, the diagnosis of metastatic ILC was made due to the fact that markers considered more specific for mammary carcinoma (GATA-3, mammaglobin, and GCDFP-15) were positive.

Our literature review has revealed three cases of breast carcinoma metastasis to the colon coexisting with colonic adenocarcinoma [4-5,20]. Two of these involved ILC and the third was NST carcinoma. In the first case, the metastatic ILC was undiagnosed until colorectal resection [4] whereas the other two cases had a known history of ILC [5,20]. The clinicopathological features of these cases are presented in Table 1. Interestingly, the patient with colonic metastasis by NST carcinoma had a history of ILC 30 years prior [20]. The lymph nodes were infiltrated by both colorectal and breast carcinoma in the two cases involving ILC [4-5]. As in our case, the primary tumor was not located by further imaging studies in one of the cases [4].

**Table 1:** Clinicopathological features in cases of breast carcinoma metastasis to the colon co-existing with colonic adenocarcinoma. Abbreviations: Adj. Therapy: adjuvant therapy; ANED: alive no evidence of disease; Ca: cancer; Chemo: chemotherapy; Conc: concomitant; CRC: colorectal carcinoma; G: grade; Horm: hormonal treatment; ILC: invasive lobular carcinoma; mo: months; NST: no special type carcinoma. *Grade is not mentioned **Patient refused treatment ***Patient was lost to follow up

Case	Year	Age	Timing	Breast Ca	Colon Ca	Adj. Therapy	Outcome (mo)
1^4^	2013	78	9 years	ILC *	CRC G2	Horm+Chemo	48 ANED
2^20^	2014	83	Conc.	NST G2	CRC G2	Horm	18 ANED
3^5^	2015	80	Conc.	ILC G2	CRC G2	Horm	4 ANED
Present case	2017	87	Conc.	ILC G2	CRC G3	**	***

There is no consensus regarding the treatment of metastatic breast cancer with intestinal involvement due to its rarity. Systemic treatment with surgery or surgery alone has been suggested to have a favorable outcome in some reports [20].

## Conclusions

While breast carcinoma colorectal metastasis is a rare event, primary colonic and metastatic breast carcinoma coexistence is extremely rare. The precise diagnosis of metastatic breast carcinoma in a colectomy specimen may be very challenging, especially in cases lacking a previous history. In such cases, immunohistochemistry usually provides a diagnostic solution. Systemic treatment has been suggested to have a favorable outcome in some reports.
